# Supplementary material for: Support measures for the next of kin who has experienced the unexpected loss of a family member to HIV/AIDS
Source: PLoS One. 2023 Apr 11;18(4):e0283903. doi: 10.1371/journal.pone.0283903 (PMC10089320; doi:10.1371/journal.pone.0283903)
Supplement: S1 File — (PDF) [file pone.0283903.s001.pdf]

Cape Peninsula
University of Technology

HEALTH AND WELLNESS SCIENCES RESEARCH ETHICS COMMITTEE (HW-REC)
Registration Number NHREC: REC- 230408-014

P.O. Box 1906 • Bellville 7535 South Africa
Symphony Road Bellville 7535
Tel: +27 21 959 6917
Email: simonsy@cput.ac.za

4 November 2019
REC Approval Reference No:
CPUT/HW-REC 2019/H2

Dear Ms Siphesihle Delani Hlophe

Re: APPLICATION TO THE HW-REC FOR ETHICS CLEARANCE

Approval was granted by the Health and Wellness Sciences-REC to Ms Siphesihle Delani Hlophe for ethical clearance on 4 November 2019. This approval is for research activities related to student research in the Department of Nursing of this Institution.

TITLE: **Self-management of youths who have lost a family member to HIV/AIDS in a comprehensive primary healthcare centre in Cape Town**

Supervisor: Prof K Jooste

Comment:

Approval will not extend beyond 5 November 2020. An extension should be applied for 6 weeks before this expiry date should data collection and use/analysis of data, information and/or samples for this study continue beyond this date.

The investigator(s) should understand the ethical conditions under which they are authorized to carry out this study and they should be compliant to these conditions. It is required that the investigator(s) complete an **annual progress report** that should be submitted to the HWS-REC in December of that particular year, for the HWS-REC to be kept informed of the progress and of any problems you may have encountered.

Kind Regards

Dr. Navindhra Naidoo
Chairperson – Research Ethics Committee
Faculty of Health and Wellness Sciences
