# Supplementary material for: Support measures for the next of kin who has experienced the unexpected loss of a family member to HIV/AIDS
Source: PLoS One. 2023 Apr 11;18(4):e0283903. doi: 10.1371/journal.pone.0283903 (PMC10089320; doi:10.1371/journal.pone.0283903)
Supplement: S2 File — (PDF) [file pone.0283903.s002.pdf]

Western Cape Government
Health

STRATEGY & HEALTH SUPPORT
Health.Research@westerncape.gov.za
tel: +27 21 483 0866: fax: +27 21 483 6058
5th Floor, Norton Rose House,, 8 Riebeek Street, Cape Town, 8001
www.capegateway.gov.za)

REFERENCE: WC_201911_032
ENQUIRIES: Dr Sabela Petros

P.O. Box 1906
Symphony Road
Bellville
7535
South Africa

For attention: MR Siphesihle Delani Hlophe

Re: Self-management of youths who have lost a family member to HIV/AIDS in a comprehensive primary healthcare centre in Cape Town

Thank you for submitting your proposal to undertake the above-mentioned study. We are pleased to inform you that the department has granted you approval for your research.

Please contact the following people to assist you with any further enquiries in accessing the following sites:

**Khayelitsha (Site B) CHC**	**Leigh Wagner**	**021 360 5228/ 5238**

Kindly ensure that the following are adhered to:

1. Arrangements can be made with managers, providing that normal activities at requested facilities are not interrupted.
2. Researchers, in accessing provincial health facilities, are expressing consent to provide the department with an electronic copy of the final feedback (**annexure 9**) within six months of completion of research. This can be submitted to the provincial Research Co-ordinator (Health.Research@westerncape.gov.za).
3. In the event where the research project goes beyond the *estimated completion* date which was submitted, researchers are expected to complete and submit a progress report (**Annexure 8**) to the provincial Research Co-ordinator (Health.Research@westerncape.gov.za).
4. The reference number above should be quoted in all future correspondence.

Yours sincerely

DR G DENICKER
ACTIVE DIRECTOR: HEALTH IMPACT ASSESSMENT
DATE: 18/12/2019
CC
