# Supplementary material for: Support measures for the next of kin who has experienced the unexpected loss of a family member to HIV/AIDS
Source: PLoS One. 2023 Apr 11;18(4):e0283903. doi: 10.1371/journal.pone.0283903 (PMC10089320; doi:10.1371/journal.pone.0283903)
Supplement: Attachment — Submitted filename: Response to Reviewers.docx [file pone.0283903.s003.docx]

**Rebuttal letter**

|  | **Reviews comment** | **Author** |
| --- | --- | --- |
| 1 | Please ensure that your manuscript meets PLOS ONE's style requirements, including those for file naming. | The manuscript was revised to meet PLOS ONE style templates as per the link provided. |
| 2 | A. Please clarify the sources of funding (financial or material support) for your study. List the grants or organizations that supported your study, including funding received from your institution. B. State what role the funders took in the study. If the funders had no role in your study, please state: “The funders had no role in study design, data collection and analysis, decision to publish, or preparation of the manuscript.” C. If any authors received a salary from any of your funders, please state which authors and which funders. | The authors received no specific funding for this work |
| 3 | Thank you for stating the following in your Competing Interests section:  “no”   D. If you did not receive any funding for this study, please state: “The authors received no specific funding for this work.” | The authors have declared that no competing interests exist.  Competing Interests has been completed online on the submission form.  The authors received no specific funding for this work. The amended statements have been included in the cover letter. |
| 4 | In your revised cover letter, please address the following prompts:  a) If there are ethical or legal restrictions on sharing a de-identified data set, please explain them in detail (e.g., data contain potentially sensitive information, data are owned by a third-party organization, etc.) and who has imposed them (e.g., an ethics committee). Please also provide contact information for a data access committee, ethics committee, or other institutional body to which data requests may be sent.  b) If there are no restrictions, please upload the minimal anonymized data set necessary to replicate your study findings as either Supporting Information files or to a stable, public repository and provide us with the relevant URLs, DOIs, or accession numbers. | There are no restrictions to accessing study data; the URL to the repository is as below and is accessible by the public:  <https://etd.cput.ac.za/handle/20.500.11838/3370>  Research Ethics Committee: Ms Nomathemba Seth, [SethN@cput.ac.za](mailto:SethN@cput.ac.za) Prof Karien Jooste [joosteka@cput.ac.za](mailto:joosteka@cput.ac.za)  Tel: +27 21 959 6523 |
| 5 | Please include your tables as part of your main manuscript and remove the individual files. Please note that supplementary tables (should remain/ be uploaded) as separate "supporting information" files. | Table included as part of the main manuscript and individual files removed. |
| 6 | Please include captions for your Supporting Information files at the end of your manuscript, and update any in-text citations to match accordingly. | Supporting Information files updated on the manuscript accordingly. |
| 7 | Please review your reference list to ensure that it is complete and correct. If you have cited papers that have been retracted, please include the rationale for doing so in the manuscript text, or remove these references and replace them with relevant current references. | Reference have been reviewed to ensure that all links and DOIs are working. |

|  | **Review Comments to the Author** | **Author** |
| --- | --- | --- |
| 1 | The range of "youth" age group should be explained and justified in the method section (for example, based on what references?) | The youth age range has been added and explained int the introduction if the manuscript |
| 2 | Some comparison statement in findings could be re-organized and written into the discussion section. | Some comparison statements in the findings were re-organized to the discussion section. |
